# Supplementary material for: Long-Term Outcomes of Breast Cancer Patients Who Underwent Selective Neck Dissection for Metachronous Isolated Supraclavicular Nodal Metastasis
Source: Cancers (Basel). 2021 Dec 29;14(1):164. doi: 10.3390/cancers14010164 (PMC8750885; doi:10.3390/cancers14010164)
Supplement: Supplementary file 1 [file cancers-14-00164-s001.zip › cancers-1457201-supplementary/Figure S1.pdf]

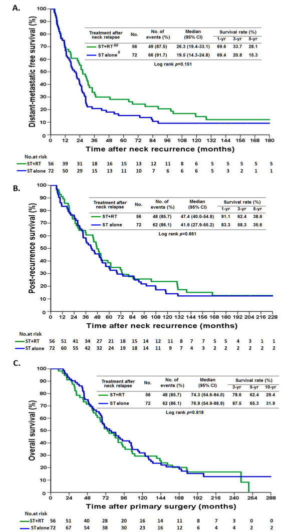

Figure S1 Kaplan-Meier curve of A, distant-metastatic free survival B, post-recurrence survival C, overall survival for miSLNM patients by radiotherpay

#ST: systemic therapy; ##RT: radiotherapy
