# Supplementary material for: Long-Term Outcomes of Breast Cancer Patients Who Underwent Selective Neck Dissection for Metachronous Isolated Supraclavicular Nodal Metastasis
Source: Cancers (Basel). 2021 Dec 29;14(1):164. doi: 10.3390/cancers14010164 (PMC8750885; doi:10.3390/cancers14010164)
Supplement: Supplementary file 1 [file cancers-14-00164-s001.zip › cancers-1457201-supplementary/Figure S2.pdf]

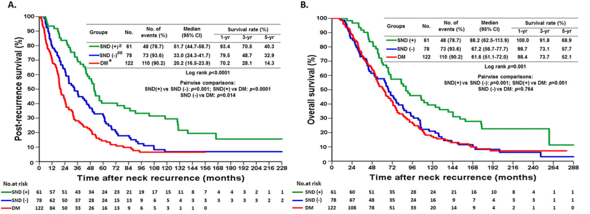

Figure S2 Kaplan-Meier curve of A, post-recurence survival B, overall survival for miSLNM and miSLNM with distant metastasis

SND (+): miSLNM with neck dissection
SND (-): miSLNM without neck dissection
* DM: miSLNM with distant metastasis
