# Supplementary material for: Long-Term Outcomes of Breast Cancer Patients Who Underwent Selective Neck Dissection for Metachronous Isolated Supraclavicular Nodal Metastasis
Source: Cancers (Basel). 2021 Dec 29;14(1):164. doi: 10.3390/cancers14010164 (PMC8750885; doi:10.3390/cancers14010164)
Supplement: Supplementary file 1 [file cancers-14-00164-s001.zip › cancers-1457201-supplementary/Figure S3.pdf]

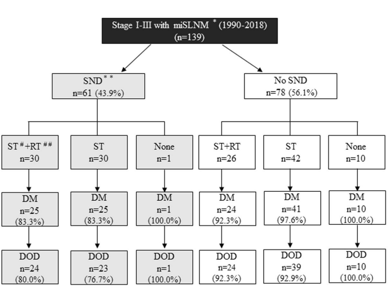

*miSLNM: metachronous isolated supraclavicular lymph node metastasis; **SND: selective neck dissection; #ST: systemic therapy; ##RT: radio therapy; DM: distant metastasis; DOD: Died of disease

Figure S3 Overview of treatment patterns and outcomes based on selective neck dissection or not
