# Supplementary material for: Long-Term Outcomes of Breast Cancer Patients Who Underwent Selective Neck Dissection for Metachronous Isolated Supraclavicular Nodal Metastasis
Source: Cancers (Basel). 2021 Dec 29;14(1):164. doi: 10.3390/cancers14010164 (PMC8750885; doi:10.3390/cancers14010164)
Supplement: Supplementary file 1 [file cancers-14-00164-s001.zip › cancers-1457201-supplementary/Tables S1.pdf]

Table S1. Univariate and Multivariate Analysis of Post-recurrence Survival after Metachronous Isolated Supraclavicular Lymph Node Metastasis

Variables		No.	Median Survival Time (Months)	95% CI * of Median	*p* Δ Value	HR	95% CI of HR	*p* Value
Initial clinical features								
Age (years)	≤40	42	45.3	24.3–66.3	0.673	–		
	>40	97	44.2	34.2–54.1				
Tumor size (cm)	≤3	92	46.9	38.6–55.2	0.397	–		
	>3	47	38.6	15.0–62.3				
Axillary involvement	Yes	105	41.6	30.9–52.2	0.118	–		
	No	34	51.4	31.2–71.6				
Estrogen receptor status	Positive	75	44.2	21.6–66.7	0.391	–		
	Negative	57	46.5	38.6–54.3				
Progesterone receptor status	Positive	69	49.0	35.5–62.5	0.690	–		
	Negative	63	44.6	33.5–55.7				
HER-2/ neu	Positive	38	46.5	38.2–54.7	0.987	–		
	Negative	48	51.4	28.1–74.8				
SBR grade	1	15	68.2	54.3–82.0	0.367	–		
	2	46	49.7	40.3–59.0				
	3	48	36.1	29.8–42.5				
Axillary level II dissection	Yes	118	44.6	38.2–51.0	0.977	–		
	No	21	32.0	20.4–43.5				
Adjuvant therapy before relapse								
Chemotherapy	Yes	124	44.6	37.5–51.7	0.717	–		
	No	15	20.2	7.6–32.8				
Hormonal therapy	Yes	73	49.0	29.4–68.6	0.255	–		
	No	66	43.9	34.2–				

				53.5				
Radiotherapy	Yes	32	44.2	13.5–74.8	0.588	–		
	No	107	44.6	33.7–55.5				
Clinical features after relapse								
Age at relapse	≤50	70	41.6	29.1–54.0	0.670	–		
	>50	69	46.9	35.8–58.1				
Clinical neck node size (cm)	≤1.3	47	43.3	31.8–54.7	0.240	–		
	>1.3	46	42.4	32.7–52.1				
Selective neck dissection	Yes	61	51.7	44.7–58.7	0.001	1		
	No	78	33.0	24.3–41.7		1.77	1.22–2.56	.003
Time interval from primary tumor surgery to neck relapse (months)	≤24	64	29.1	21.7–36.5	<0.0001	2.07	1.44–2.98	<.0001
	>24	75	51.6	41.0–62.3		1		
Chemotherapy	Yes	113	44.2	34.8–53.6	0.568	–		
	No	26	44.2	0.1–88.9				
Hormonal therapy **	Yes	78	52.5	36.5–68.5	0.053	–		
	No	12	27.7	0.1–68.9				
Radiotherapy	Yes	57	47.4	40.5–54.2	0.496	–		
	No	82	41.0	30.1–52.0				

95% CI: 95% confidence interval; Δlog rank test; *HR: hazard ratio
** Select primary tumor or neck tumor with ER and/or PR (+) cases
