# Supplementary material for: Long-Term Outcomes of Breast Cancer Patients Who Underwent Selective Neck Dissection for Metachronous Isolated Supraclavicular Nodal Metastasis
Source: Cancers (Basel). 2021 Dec 29;14(1):164. doi: 10.3390/cancers14010164 (PMC8750885; doi:10.3390/cancers14010164)
Supplement: Supplementary file 1 [file cancers-14-00164-s001.zip › cancers-1457201-supplementary/Tables S2.pdf]

Table S2. Univariate and Multivariate Analysis of Overall Survival for miSLNM Patients

Variables		No.	Median Survival Time (Months)	95% CI * of Median	*p* Δ Value	HR	95% CI of HR	*p* Value
Initial clinical features								
Age (years)	≤40	42	68.0	52.7–83.4	0.927	–		
	>40	97	82.6	64.3–101.0				
Tumor size (cm)	≤3	92	76.9	61.9–91.9	0.302	–		
	>3	47	66.3	54.7–77.9				
Axillary involvement	Yes	105	88.2	50.9–125.5	0.140	–		
	No	34	67.6	54.5–80.7				
Estrogen receptor status	Positive	75	105.2	82.8–127.7	0.081	–		
	Negative	57	70.2	60.4–79.9				
Progesterone receptor status	Positive	69	88.8	47.1–130.4	0.408	–		
	Negative	63	73.1	58.0–88.2				
HER-2/ neu	Positive	38	67.1	59.4–74.8	0.159	–		
	Negative	48	105.2	89.9–120.5				
SBR grade	1	15	94.3	72.9–115.6	0.231	–		
	2	46	70.2	50.8–89.5				
	3	48	65.1	416–88.5				
Axillary level II dissection	Yes	118	74.3	60.6–87.7	0.991	–		
	No	21	64.4	13.9–115.0				
Adjuvant therapy before relapse								
Chemotherapy	Yes	124	76.3	61.7–91.0	0.674	–		
	No	15	67.6	38.0–97.2				
Hormonal therapy	Yes	73	88.8	44.9–132.7	0.127	–		
	No	66	70.2	59.2–81.2				
Radiotherapy	Yes	32	67.6	60.5–74.7	0.166	–		
	No	107	76.9	55.3–98.5				
Clinical features after relapse								
Age at relapse	≤50	70	68.0	57.9–78.1	0.387	–		
	>50	69	85.1	62.5–107.6				
Clinical neck node size (cm)	≤1.3	47	66.3	55.6–77.0	0.196	–		
	>1.3	46	73.1	56.3–89.9				
Selective neck dissection	Yes	61	88.2	62.5–113.9	0.001	1		
	No	78	67.2	56.7–777		1.77	1.22–2.55	0.003
Time interval from primary	≤24	64	43.5	35.0–51.9	<0.0001	3.54	2.44–	<0.0001

tumor surgery to neck							5.16
relapse (months)	>24	75	106.7	97.6–116.0		1	
Chemotherapy	Yes	113	73.1	59.7–86.5	0.442	–	
	No	26	82.6	19.0–146.2			
Hormonal therapy **	Yes	78	84.8	50.7–119.0	0.162	–	
	No	12	62.4	0.1–145.8			
Radiotherapy	Yes	57	74.3	54.2–94.4	0.945	–	
	No	82	73.1	57.2–89.0			

[#] 95% CI: 95% confidence interval; Δlog rank test; *HR: hazard ratio
** Select primary tumor or neck tumor with ER and/or PR (+) cases
